# System-level mapping of *Escherichia coli* response regulator dimerization with FRET hybrids

**DOI:** 10.1111/j.1365-2958.2008.06355.x

**Published:** 2008-07-15

**Authors:** Rong Gao, Yuan Tao, Ann M Stock

**Affiliations:** 1 Center for Advanced Biotechnology and Medicine. UMDNJ-Robert Wood Johnson Medical SchoolPiscataway, NJ 08854, USA; 2 Department of Biochemistry, UMDNJ-Robert Wood Johnson Medical SchoolPiscataway, NJ 08854, USA; 3 Howard Hughes Medical Institute, UMDNJ-Robert Wood Johnson Medical SchoolPiscataway, NJ 08854, USA

## Abstract

Two-component signal transduction, featuring highly conserved histidine kinases (HKs) and response regulators (RRs), is one of the most prevalent signalling schemes in prokaryotes. RRs function as phosphorylation-activated switches to mediate diverse output responses, mostly via transcription regulation. As bacterial genomes typically encode multiple two-component proteins for distinct signalling pathways, the sequence and structural similarities of RR receiver domains create significant challenges to maintain interaction specificity. It is especially demanding for members of the OmpR/PhoB subfamily, the largest RR subfamily, which share a conserved dimerization interface for phosphorylation-mediated transcription regulation. We developed a strategy to investigate RR interaction by analysing Förster resonance energy transfer (FRET) between cyan fluorescent protein (CFP)- and yellow fluorescent protein (YFP)-fused RRs *in vitro*. Using the *Escherichia coli* RR PhoB as a model system, we were able to observe phosphorylation-dependent FRET between fluorescent protein (FP)–PhoB proteins and validated the FRET method by determining dimerization affinity and dimerization-coupled phosphorylation kinetics that recapitulated values determined by alternative methods. Further application of the FRET method to all *E. coli* OmpR/PhoB subfamily RRs revealed that phosphorylation–activated RR interaction is indeed a common theme for OmpR/PhoB subfamily RRs and these RRs display significant interaction specificity. Weak hetero-pair interactions were also identified between several different RRs, suggesting potential cross-regulation between distinct pathways.

## Introduction

Cells have evolved extraordinarily complex and sophisticated signalling mechanisms to monitor and adapt to their environments. The capability of processing information from diverse internal and external cues is crucial to the fitness and survival of cells. As for numerous different signalling pathways, it is a common theme that certain domains with similar core structures and conserved functions, such as phosphorylation, methylation and protein recognition, have been repeatedly exploited as the building blocks for signalling proteins. The modular design of these signalling protein families allows the coupling of arrays of different input and output domains with the central conserved core function to form distinct and versatile pathways responding to a wide variety of signals. Conversely, as the sequence and structural similarity of the conserved core domains may be advantageous for interactions and communications between different signalling pathways, it also creates significant challenges to maintain the signalling specificity and fidelity among large numbers of similar signalling proteins within the same family. Thus, it becomes increasingly important to understand the interactions and specificity within a single family, which could lead to insights regarding the evolution of structure–function relationships of these signalling proteins as well as the discovery of novel cross-regulation between different pathways.

**Fig. 1:**
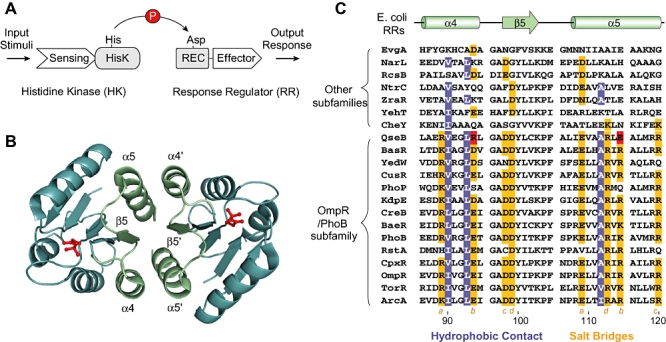
Two-component signal transduction and the OmpR/PhoB subfamily of response regulators. A. Schematic outline of a typical two-component system. The pathway features transfer of a phosphoryl group (red) between the conserved histidine kinase and receiver domains (HisK and REC, grey). Sensing of input stimuli by the HK modulates the kinase or phosphatase activity and regulates the phosphorylation level of the RR. The phosphorylated RR elicits the output response through the effector domain. B. Dimer structure of the PhoB receiver domain (PDB ID: 1ZES). The receiver domain has a conserved (βα)_5_ fold (teal blue) and OmpR/PhoB subfamily RRs appear to share a conserved dimeric structure once phosphorylated. The non-covalent phosphoryl analogue beryllofluoride (
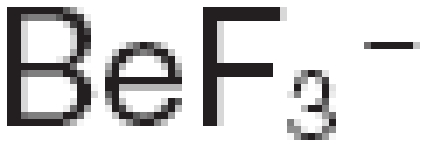
) co-ordinates to the conserved aspartate residue (red), allosterically perturbing the α4-β5-α5 surface (green) and promoting dimerization. C. Sequence alignment of the α4-β5-α5 region of *E. coli* RRs. Full-length RR sequences are aligned by clustalx (Thompson *et al*., 1997) and only the α4-β5-α5 region is shown. Sequence numbering is for ArcA. RRs include all *E. coli* OmpR/PhoB subfamily members and some representatives from other subfamilies. Among the highly conserved residues within the OmpR/PhoB subfamily are the highlighted residues that are involved in intermolecular interactions: hydrophobic contacts (blue); charged residues for salt bridge formation (orange). The pairing of charged residues is labelled by four pairs of letters *a, b*, *c* and *d*. A red highlight represents a pair of residues that are not conserved but could still complement each other with reversed charges. All these highlighted residues are not well conserved in RRs from other families.

Two-component signal transduction is one of the most prevalent signalling schemes in bacteria, participating in various cell regulatory tasks such as chemotaxis, nutrition utilization, virulence, quorum sensing and cell cycle regulation. Typical two-component systems consist of two major families of signalling proteins, sensor histidine protein kinases (HKs) and response regulators (RRs) (see reviews by Stock *et al*., 2000; Gao *et al*., 2007). HKs share a kinase domain that catalyses autophosphorylation at a conserved histidine residue, while the conserved receiver domain of RRs catalyses transfer of a phosphoryl group from the phosphoHis of the HK to one of its own aspartate residues (Fig. 1A). Signal perception by the sensing domain of the HK regulates the kinase activity and, in some bifuncitional HKs, the phosphatase activity as well, to mediate the phosphorylation level of its cognate RR. The phosphorylated RR functions as the ultimate control element that modulates the activity of its effector domain and elicits the particular response. The sensing domain of HKs and the effector domain of RRs show great diversity, which allows the utilization of the same His–Asp phosphotransfer scheme in distinct HK–RR pairs to couple diverse input stimuli, such as nutrients, redox state, osmolarity and antibiotics, to an equally diverse range of output responses, most frequently through transcriptional regulation, but also by mediation of protein–protein interactions and enzyme activities.

Most sequenced bacterial genomes encode multiple two-component proteins, with the number positively correlating with the genome size (Galperin, 2005; Ulrich and Zhulin, 2007). There are 30 HKs and 32 RRs in *Escherichia coli*, and the total number of two-component proteins exceeds 200 in *Myxococcus xanthus* and some cyanobacteria species. Both lineage-specific expansion and horizontal gene transfer are thought to contribute to the evolution of diverse two-component pathways that allow bacteria to adapt to complex environments (Alm *et al*., 2006). The presence of many paralogous HK/RR proteins in the same cell requires individual pathways to be insulated from one another to ensure signal transmission fidelity and avoid detrimental cross-talk despite their highly similar sequences and structures. This is especially demanding for the OmpR/PhoB subfamily of RRs; they share not only similar structures for individual domains but also a common active dimer state that is believed to be conserved within the subfamily (Bachhawat *et al*., 2005; Toro-Roman *et al*., 2005a,b).

Response regulators are classified into different subfamilies according to their effector domains. The OmpR/PhoB subfamily of RRs is the largest subfamily, characterized by a winged helix–turn–helix effector domain for DNA binding. They account for ∼30% of all RRs and half of the RRs possessing a DNA-binding domain (Galperin, 2006). Fourteen out of 32 RRs in *E. coli* belong to this subfamily. OmpR and PhoB, the eponymous members of this subfamily, are well-studied RRs responsible for osmoregulation and phosphate assimilation in *E. coli* respectively (Pratt and Silhavy, 1995; Wanner, 1996). The RR receiver domain is an α/β domain with a conserved activation mechanism in which phosphorylation of the aspartate residue allosterically affects a distant surface, primarily the α4-β5-α5 face, to mediate inter- or intra-protein interactions. The sequence of the α4-β5-α5 region is highly conserved within the OmpR/PhoB subfamily with a ∼60% sequence identity compared with the 20–30% identity typically observed over the entire length of RRs. This represents a significant difference that distinguishes the OmpR/PhoB subfamily from other subfamilies, such as the NtrC/DctD and the NarL/FixJ subfamilies. Structural characterization of a few RRs from the OmpR/PhoB subfamily reveals that the α4-β5-α5 face is the dimerization interface (Fig. 1B) with a common set of hydrophobic and charged residues involved in van der Waals contact and salt bridges (Fig. 1C) (Toro-Roman *et al*., 2005a).

The sequence conservation of the contacting residues suggests a similar dimerization interface for most OmpR/PhoB subfamily members. Given the prevalence of this subfamily in bacteria, it is important to question the specificity of the dimerizing interactions among subfamily members and whether RR heterodimers can form. While unproductive RR heterodimers could be problematic for the fidelity of signal transmission, particular heterodimer pairs might be valuable to the integration of different pathways via transcription of a distinct set of heterodimer-regulated genes for a co-ordinated response to complex environmental conditions. In contrast to eukaryotic signalling pathways in which the regulatory roles of both homodimers and heterodimers are well documented, e.g. the receptor tyrosine kinase family and the nuclear hormone receptor superfamily (Schlessinger, 2000; Aranda and Pascual, 2001; de Lera *et al*., 2007), RR heterodimers are rarely reported (Knutsen *et al*., 2004) and the dimerization specificity of RRs is uncharacterized. Here we report development of a strategy to investigate RR dimerization via measuring the Förster resonance energy transfer (FRET) between cyan fluorescent protein (CFP)- and yellow fluorescent protein (YFP)-fused RRs *in vitro*. The FRET method allowed us to systematically analyse all possible protein–protein interactions among 14 OmpR/PhoB subfamily members from *E. coli*. We demonstrate that these RRs show significant dimerization specificity in spite of the conserved residues at the dimerization interface. Further, interactions between several different RRs do occur, suggesting potential cross-regulation between some two-component pathways.

## Results

### Functional characterization of fluorescent protein-fused PhoB

**Fig. 2:**
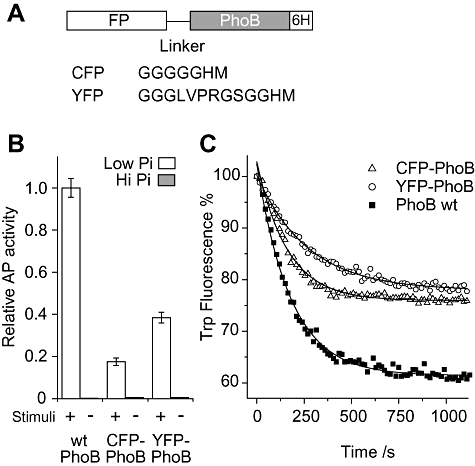
Alkaline phosphatase (AP) assays and *in vitro* phosphorylation of FP–PhoB hybrids. A. Diagram of FP–PhoB proteins. Different flexible linkers are engineered between PhoB and different FPs. For YFP–PhoB, an arginine residue in the linker makes it susceptible to trypsin digestion. All FP–PhoB proteins have a His_6_ tag for purification. B. Expression of AP. A *phoB* deletion strain carrying either pRG20 (CFP–PhoB) or pRG94 (YFP–PhoB) and the wild-type strain BW25113 were assayed for AP activity under low-phosphate (Pi) (white) and high-Pi (grey) conditions. FP–PhoB hybrids were induced by IPTG to an expression level of protein comparable to that of wild-type PhoB. The data are from three replicates. Protein expression levels were confirmed to be comparable by Western blot analyses (data not shown). C. Tryptophan fluorescence quenching upon phosphorylation. Tryptophan fluorescence of individual PhoB proteins (2 μM) was monitored at the emission wavelength of 345 nm with the excitation at 295 nm. Proteins include CFP–PhoB (open triangle), YFP–PhoB (open circle) and PhoB (solid squares). These proteins were mixed with 20 mM phosphoramidate in the reaction buffer and the phosphorylation was initiated by addition of MgSO_4_ to a final concentration of 5 mM. Solid lines represent the fitted exponential decay curves. Three repeated experiments gave the average rate constants as: CFP–PhoB, 6.6 ± 0.6 × 10^−3^ s^−1^; YFP–PhoB, 3.5 ± 0.4 × 10^−3^ s^−1^; PhoB, 5.6 ± 0.6 × 10^−3^ s^−1^.

PhoB, a representative member of the OmpR/PhoB subfamily, is one of the most extensively studied RRs in *E. coli* and was chosen to establish the FRET method for RR interactions. Paired with the HK PhoR, PhoB responds to phosphate-limiting conditions and regulates a large set of genes involved in phosphate uptake and utilization of alternative phosphorus sources, including *phoA*, which encodes an alkaline phosphatase (AP) (Wanner, 1993; Lamarche *et al*., 2008). As previously demonstrated, assays of AP activity showed minimal expression of *phoA* under high phosphate conditions, while low phosphate in the media activated PhoB to promote *phoA* expression (Fig. 2). CFP and YFP were fused at the N-terminus of PhoB with flexible linkers (Fig. 2A). When expressed at similar levels as wild-type PhoB in an *E. coli phoB* deletion strain, both fluorescent protein (FP)-fused PhoB proteins activated *phoA* expression, although the levels of AP activity were lower compared with the corresponding level in the wild-type strain (Fig. 2B). The lower *phoA* expression might result from less efficient phosphorylation by the kinase, weaker DNA binding or non-optimal interaction with RNA polymerase compared with wild-type PhoB. Nevertheless, both FP–PhoB proteins regulate *phoA* expression in response to phosphate limitation, functionally complementing the *phoB* deletion.

It has been shown that phosphorylation of PhoB decreases its intrinsic tryptophan fluorescence and the kinetics of fluorescence quenching reflect the phosphorylation kinetics (McCleary, 1996). Both CFP–PhoB and YFP–PhoB showed the characteristic tryptophan fluorescence quenching upon addition of the small-molecule phosphodonor phosphoramidate (Fig. 2C). The difference in the percentage of fluorescence change is likely due to the different number of tryptophan residues in PhoB (3) and FP–PhoB proteins (5). Moreover, the fluorescence quenching kinetics can be fitted to a pseudo first-order rate equation with the apparent rate constant of 0.0066 s^−1^ for CFP–PhoB and 0.0035 s^−1^ for YFP–PhoB, close to that of PhoB alone (0.0056 s^−1^), showing that both FP–PhoB proteins can be phosphorylated by phosphoramidate *in vitro* similarly as PhoB.

### Analysis of phosphorylation-dependent PhoB dimerization by FRET

**Fig. 3:**
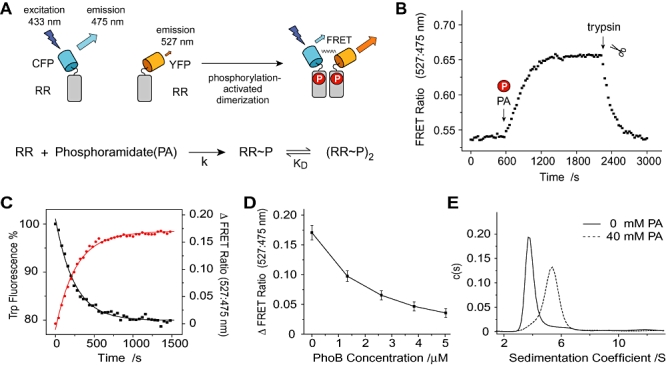
FRET analyses of FP–PhoB dimerization. A. FRET scheme of phosphorylation-dependent dimerization. Phosphorylation promotes dimerization and enables FRET, which is characterized by a decrease of cyan emission and an increase of yellow emission. The rate of FRET increase depends on the rates of phosphorylation and dimerization. B. Phosphorylation-activated FRET of FP–PhoB hybrids. Fluorescence at 475 nm and 527 nm were recorded to calculate the FRET ratio. The clock started after addition of YFP–PhoB protein to the CFP–PhoB solution and the final concentrations are 0.75 μM for CFP–PhoB and 1.25 μM for YFP–PhoB respectively. Phosphorylation was initiated by adding MgSO_4_ at ∼600 s and FRET was disrupted by adding 1 μl of trypsin to a final concentration of 5 μg ml^−1^ at ∼2250 s to release YFP from the dimer. C. Comparison of rates for tryptophan fluorescence quenching (black) and FRET increase (red). Cyan, yellow and tryptophan fluorescence were followed for a FP–PhoB mixture containing 0.6 μM CFP–PhoB and 2.5 μM YFP–PhoB. The value of FRET ratio change upon phosphorylation is used to reflect the extent of FRET. Solid lines represent the fitted curves and the rate constants are 4.1 × 10^−3^ s^−1^ for the quenching of tryptophan fluorescence and 3.9 × 10^−3^ s^−1^ for the FRET ratio increase. D. Competition of the FP–PhoB interaction with PhoB. CFP–PhoB (0.6 μM) and YFP–PhoB (2.5 μM) were phosphorylated in the presence of 20 mM PA. PhoB was subsequently titrated into the mixture to reach the indicated concentrations and fluorescence was measured at 600 s intervals. The change of FRET ratio was calculated by subtracting the ratio of the initial non-phosphorylated FP–PhoB pairs. Error bars represent the standard deviations from three independent experiments. E. Continuous sedimentation coefficient distribution [*c*(*s*)] of the mixture of 5.5 μM CFP–PhoB and 3.0 μM YFP–PhoB. Sedimentation velocity profiles were collected for phosphorylated (dotted) and unphosphorylated (solid) samples using absorbance optics at 514 nm, corresponding to the YFP absorbance. Nearly identical *c*(*s*) distribution was obtained for the CFP characteristic absorbance at 433 nm (Fig. S2).

The interaction between CFP- and YFP-fused PhoB proteins is measured by the distance-dependent energy transfer from the excited donor CFP to the acceptor YFP. A higher ratio of yellow (527 nm) to cyan emission (475 nm) indicates more interacting CFP/YFP pairs (Fig. 3A). As shown in Fig. 3B, the FRET ratio (emission ratio 527:475 nm) remained steady before addition of the phosphodonor phosphoramidate and phosphorylation promoted FRET between CFP-/YFP-fused PhoB with the FRET ratio reaching a maximum in 10 min. Cleaving the trypsin-susceptible linker between YFP and PhoB separated YFP from interacting PhoB proteins, and quickly returned the FRET ratio to the pre-phosphorylation level (Fig. 3B and Fig. S1). The similar FRET ratio for unphosphorylated and cleaved pairs suggests that there is little PhoB dimerization in the absence of phosphorylation at the experimental concentration (2.7 μM), which is significantly below the reported dissociation constant (*K*_D_: 378 μM) for unphosphorylated PhoB (Mack, 2008). Furthermore, the FRET ratio increased at a parallel rate as the quenching of tryptophan fluorescence with comparable apparent rate constants, 0.0039 s^−1^ for the FRET ratio change and 0.0041 s^−1^ for phosphorylation-induced tryptophan fluorescence quenching (Fig. 3C). This reveals phosphorylation as the rate-limiting step and indicates that the rate of FRET ratio change can be used to follow phosphorylation kinetics.

Phosphorylated free PhoB proteins successfully competed for interaction with FP–PhoB fusions and reduced the number of CFP/YFP pairs, as illustrated by the decreased FRET ratio when PhoB was added (Fig. 3D). Hence the FP–PhoB interaction reported by FRET appears reversible and specific to PhoB. To examine the oligomer species formed by FP–PhoB proteins, sedimentation velocity (SV) analyses were performed using an analytical ultracentrifuge. Continuous sedimentation distribution [*c*(*s*)] of the unphosphorylated FP–PhoB mixture featured a major peak at a sedimentation coefficient of 3.7 S, corresponding to a molecular weight (M.W.) of ∼54 kDa that is close to the M.W. for a FP–PhoB monomer (55 kDa). The phosphorylated mixture exhibited a peak at a larger sedimentation coefficient of 5.4 S with a corresponding M.W. of ∼100 kDa. The observation of a single peak with a M.W. slightly smaller than the 110 kDa M.W. for a FP–PhoB dimer is consistent with a fast monomer–dimer equilibrium for phosphorylated FP–PhoB proteins (Fig. 3E and Fig. S2). The absence of any significant peak at a higher sedimentation coefficient again proves that no higher-order irreversible aggregates formed after phosphorylation.

**Fig. 4:**
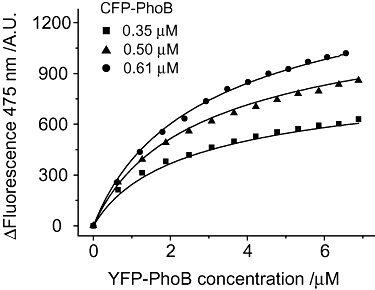
*K*_D_ determination for FP–PhoB dimerization. A mixture of phosphorylated YFP–PhoB (30 μM) and CFP–PhoB at indicated concentrations (squares, 0.35 μM; triangles, 0.5 μM; circles, 0.61 μM) were titrated into the same concentration of CFP–PhoB and the changes in CFP fluorescence at 475 nm were measured. Simultaneous fitting of all data (solid lines) gives a *K*_D_ of 4.4 μM for FP–PhoB dimerization.

Given a monomer–dimer equilibrium for FP–PhoB, the concentration of the FRET-capable CFP–/YFP–PhoB dimers can be expressed as a function of the dimerization *K*_D_ and the concentrations of CFP–PhoB and YFP–PhoB (see *Experimental procedures*). Because the decrease of cyan fluorescence (475 nm) upon phosphorylation is directly proportional to the concentration of the FRET-capable dimers, the cyan fluorescence under different initial FP–PhoB concentrations was measured to determine the *K*_D_ of FP–PhoB interaction (Fig. 4). The *K*_D_ was estimated to be 4.4 μM for FP–PhoB, similar to the *K*_D_ of 5.1 μM for PhoB determined by analytical ultracentrifugation (Mack, 2008). Therefore, FP–PhoB proteins display a similar dimerization affinity as PhoB and the FRET method is validated for investigation of RR dimerization.

### OmpR dimerization

**Fig. 5:**
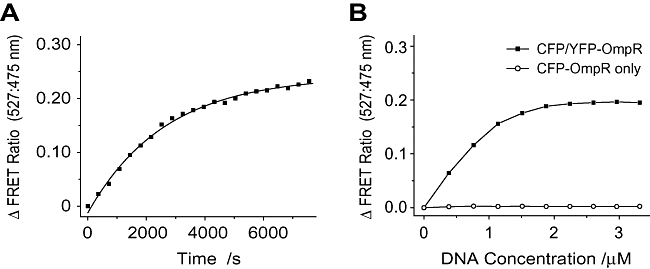
FRET analyses of FP–OmpR interaction. A. Phosphorylation-activated FRET of FP–OmpR hybrids. Equal concentrations (2 μM each) of CFP–OmpR and YFP–OmpR were phosphorylated by phosphoramidate. The fitted curve (solid line) indicates a rate constant of 0.38 × 10^−3^ s^−1^. B. Effect of DNA on FRET between FP–OmpR hybrids. C1 DNA was titrated into the mixture of 2 μM CFP–OmpR and 2 μM YFP–OmpR (solid squares) or 2 μM CFP–OmpR only (open circles) in the absence of phosphorylation.

OmpR, another representative of the OmpR/PhoB subfamily, reciprocally regulates two porin genes, *ompC* and *ompF*, in response to environmental osmolarity sensed by the cognate HK EnvZ. The dimerization of FP–OmpR was analysed in detail by the FRET method. When FP–OmpR mixtures were phosphorylated by phosphoramidate, the FRET ratio increased (Fig. 5A), but with slower phosphorylation kinetics than FP–PhoB. Addition of a C1 DNA oligonucleotide duplex containing two OmpR-binding half-sites from the *ompC* promoter greatly increased the FRET ratio even in the absence of phosphorylation (Fig. 5B), consistent with earlier reports that DNA binding stimulates OmpR dimerization (Harlocker *et al*., 1995; Maris *et al*., 2005). This dimerization could result from simultaneous binding of two OmpR molecules to the same DNA fragment. Interestingly, the FRET ratio did not decrease after further addition of C1 DNA over the stoichiometric amount, suggesting that the binding of OmpR to C1 DNA is highly cooperative, which agrees with the observation that OmpR binds DNA as dimers (Harlocker *et al*., 1995; Yoshida *et al*., 2006).

### FRET between FP–RR pairs from the *E. coli* OmpR/PhoB subfamily

**Fig. 6:**
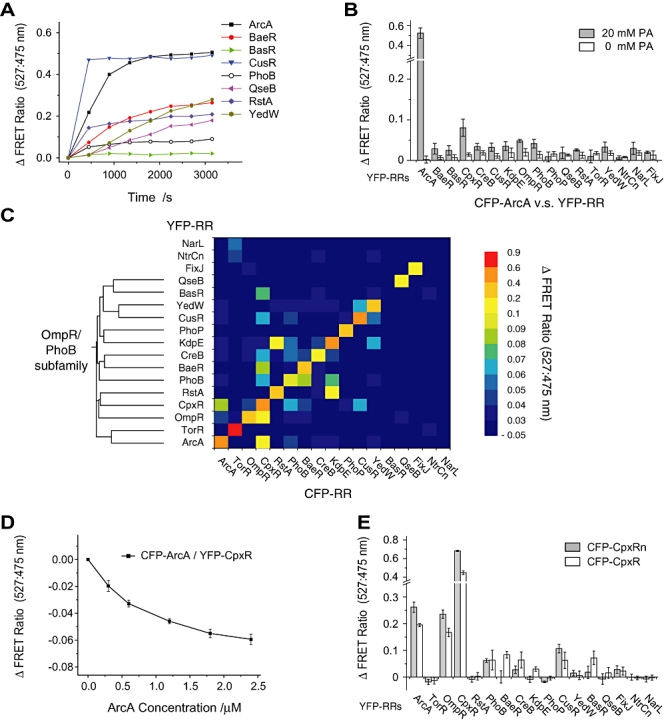
Systematic analyses of FRET between FP–RRs. Phosphorylation was initiated by addition of MgSO_4_ into the mixture of 20 mM phosphoramidate, 0.6 μM CFP–RR and 2.5 μM YFP–RR. Fluorescence was measured with a plate reader every 450 s for eight times and the FRET ratio changes at the last time point (3150 s) were used to evaluate the FRET for different FP–RR pairs. Tested RRs include all *E. coli* OmpR/PhoB subfamily RRs and three members of other subfamilies, *E. coli* NarL, the receiver domain of *E. coli* NtrC (NtrCn) and *Sinorhizobium meliloti* FixJ. A. Time-dependent FRET of RR homo-pairs. Only some of the homo-pairs are shown (refer to Fig. S3 for the rest). B. FRET between CFP–ArcA and all other YFP–RRs in the presence (grey) or absence (white) of phosphorylation. The data are from four independent experiments and the error bars indicate the standard deviations. C. FRET between phosphorylated RR pairs. Each coloured square represents one pair of FP–RRs and the diagonal squares from left bottom to right top show the FRET between homo-pairs. The colour of each square indicates the value of FRET ratio change as illustrated by the colour map on the right. The colour map is not continuous linearly in order to display and highlight the pairs with intermediate FRET levels but significantly above the background (0.05–0.2). The data are from four independent experiments while the standard deviations and FRET between non-phosphorylated RRs are shown in Fig. S3. A phylogenetic tree of the *E. coli* OmpR/PhoB subfamily RRs is shown on the left to indicate the sequence similarity between individual RRs. The phylogenetic tree was generated by clustalx from the alignment of full-length RR sequences (Thompson *et al*., 1997). D. Competition of ArcA with the interaction between CFP–ArcA and YFP–CpxR. ArcA protein was titrated into the sample containing phosphorylated CFP–ArcA (0.6 μM) and YFP–CpxR (2.5 μM) followed by the measurement of fluorescence with a fluorometer. The decrease of FRET ratio was calculated by subtracting the ratio of phosphorylated pairs without ArcA present. E. Interactions of full-length CpxR (CFP–CpxR, white) and the N-terminal receiver domain of CpxR (CFP–CpxRn, grey) with different RRs. Protein concentrations are 0.6 μM CFP–RR and 2.5 μM YFP–RR and fluorescence was measured with the fluorescence plate reader. As the yellow to cyan ratio of CFP–CpxR and CFP–CpxRn decreased upon phosphorylation, all the FRET ratios were corrected for this decrease as shown in Fig. S4.

FRET between FP-fused PhoB and OmpR clearly reported phosphorylation-induced RR dimerization. Therefore we sought to characterize the interaction specificity by examining FRET between all pairs of *E. coli* OmpR/PhoB RR subfamily members. CFP and YFP were fused to all 14 subfamily members and FRET between purified proteins was analysed using a fluorescence plate reader. All but one homo-pair (CFP–BasR/YFP–BasR) from the OmpR/PhoB RR subfamily showed increases of FRET ratio upon phosphorylation, while NarL and the receiver domain of NtrC (NtrCn), two RRs from other families, did not have any significant FRET (Fig. 6A and Fig. S3). The extent of FRET varied greatly from protein to protein, which may result from differences in interaction affinities, oligomeric states of phosphorylated RRs, levels of RR phosphorylation or positioning of FPs relative to RRs. The rate of FRET increase also varied, with the ratio reaching a maximum in less than 500 s for CusR and still increasing after ∼3000 s for QseB and YedW, reflecting different kinetics of RR phosphorylation.

The changes in FRET ratio after ∼55 min of phosphorylation were used to compare the strength of interaction between homo- and hetero-pairs of RRs. As shown in Fig. 6B, for CFP–ArcA, the highest FRET occurred when mixed with YFP–ArcA while a modest but significant FRET ratio increase was evident for YFP–CpxR. The other hetero-pairs with CFP–ArcA showed minimal changes in FRET ratio that were not greatly different from those of unphosphorylated pairs, indicating a highly specific ArcA interaction with little cross-talk to other RRs. Notably, YFP–ArcA exhibits no significant FRET in most hetero-pairs but an intermediate FRET signal with CFP–CpxR (Fig. 6C), which mirrors the pattern of interactions between CFP–ArcA and other YFP–RRs. Moreover, titration of free ArcA protein into the CFP–ArcA/YFP–CpxR mix reduced the FRET ratio significantly, demonstrating the reversibility and specificity of ArcA/CpxR interaction (Fig. 6D).

The trend of homodimerization specificity holds for all members of the *E. coli* OmpR/PhoB subfamily; FRET was the strongest for homo-pairs and there was little interaction between most hetero-pairs of RRs (Fig. 6C and Fig. S3). In a given hetero-pair, the formation of the CFP–RR homodimer or the YFP–RR homodimer is favoured over the formation of the CFP–RR/YFP–RR heterodimer due to the preference for an RR to interact with itself. Nevertheless, intermediate changes of the FRET ratio were still observed for a limited number of hetero-RR pairs, including reciprocal pairs such as ArcA/CpxR, PhoB/CpxR, CusR/CpxR, KdpE/RstA and YedW/CusR, as well as lone pairs such as CFP–CpxR/YFP–OmpR, CFP–CpxR/YFP–BaeR, CFP–CpxR/YFP–CreB, CFP–CpxR/YFP–BasR, CFP–BaeR/YFP–PhoB, CFP–KdpE/YFP–PhoB and CFP–YedW/YFP–KdpE. FRET signals in these hetero-pairs were generally much weaker than their respective homo-pairs and unequal concentrations of CFP–RR and YFP–RR may result in the observation of small FRET signals in only one pair and not in the reciprocal one.

CFP–CpxR is distinguished from other FP–RR hybrids as fusion of CpxR alters the emission spectrum of CFP. However the alteration was not observed for YFP–CpxR or the mixture of free CpxR and CFP. Further, phosphorylation shifts the emission peak of CFP–CpxR and results in a decreased ratio of 527:475 nm upon phosphorylation (Fig. S4). Therefore, the decreased FRET ratio of the CFP–CpxR/YFP pair was used to correct for the FRET ratio changes of all other pairs. Interestingly, weak FRET was suggested between CFP–CpxR and multiple RRs, such as YFP–ArcA, YFP–PhoB and YFP–CusR, which correlates with the FRET for those reciprocal pairs, CFP–ArcA/YFP–CpxR, CFP–PhoB/YFP–CpxR and CFP–CusR/YFP–CpxR (Fig. 6C and Fig. S4). The C-terminal DNA-binding domain does not appear to be essential for CpxR to interact with ArcA, OmpR, PhoB, CusR and itself, because the CFP-fused N-terminal receiver domain of CpxR displayed equal or even stronger FRET with these RRs than the full-length CpxR (Fig. 6E). Meanwhile, deletion of the DNA-binding domain completely abolished the FRET in some other pairs, such as CpxR/BaeR, suggesting that the DNA-binding domain can also play a role in interaction of some hetero-pairs.

## Discussion

### FRET analysis of RR dimerization

Protein–protein interaction is crucial for the connectivity of signalling pathways within a regulatory network. A relatively small number of protein folds and families are repeatedly used in various signalling tasks, demanding specific interactions among many structurally and functionally similar proteins. One such example in prokaryotic signalling is the prevalent OmpR/PhoB subfamily of RRs. They are believed to share a conserved dimerization interface, which is challenging for interaction specificity and also offers opportunity for beneficial connections to integrate signalling pathways. In this study we developed a FRET-based strategy to characterize the interactions at a system level within the OmpR/PhoB subfamily of RRs.

Phosphorylation-dependent FRET has been successfully observed between CFP- and YFP-fused RR pairs. Binding to the target DNA sequence also promoted the interaction of FP–OmpR pairs. Previously, the dimerization of OmpR in the absence of DNA was reported only in cross-linking studies and was not seen in other methods such as gel filtration and light scattering (Harlocker *et al*., 1995; Maris *et al*., 2005), implying the transient nature of OmpR interaction. The FRET method is apparently effective in capturing this weak OmpR interaction. Moreover, the rate of FRET change has been shown to reflect phosphorylation kinetics, which might be useful in future investigations of time-dependent signalling and RR phosphorylation *in vivo* by fluorescence imaging of bacterial cells.

About 60% of RRs have a C-terminal DNA-binding domain and phosphorylation-mediated dimerization or oligomerization is generally considered important for transcription regulation (Stock *et al*., 2000; Galperin, 2006). Yet not all FP–RR pairs showed significant FRET upon phosphorylation. It has been demonstrated that phosphorylation of the nitrogen regulator NtrC, a member of the NtrC/DctD RR subfamily, causes the receiver domain from one subunit to interact with the ATPase domain of a second subunit for oligomerization and the isolated receiver domain of NtrC remains monomeric after phosphorylation (Kern *et al*., 1999; De Carlo *et al*., 2006). Thus it is not surprising that no FRET occurred between FP-fused receiver domains of NtrC as the interaction partner ATPase domain is absent. Dimerization of the nitrate/nitrite RR NarL has only been observed in crystal structures (Baikalov *et al*., 1998; Maris *et al*., 2002) while FixJ, the RR involved in nitrogen fixation in *Sinorhizobium meliloti*, belongs to the same subfamily as NarL and has been shown biochemically to dimerize upon phosphorylation (Da Re *et al*., 1999a). Accordingly, our FRET analyses revealed no interaction for NarL but significant FRET for FixJ. Therefore, although RR multimerization is usually deemed necessary for transcription regulation, the actual interaction mechanism differs and some RR proteins do not have a strong interaction in the absence of DNA.

In contrast, phosphorylation increased FRET for almost all *E. coli* OmpR/PhoB subfamily RRs except BasR. The absence of any FRET for FP–BasR could result from low affinity of dimerization or insufficient phosphorylation. SV analysis of YFP–BasR at a higher concentration (6.9 μM) demonstrated a species with the M.W. of a dimer in addition to the monomer species (Fig. S2). The presence of two peaks instead of a single peak suggested a slow rate of exchange between two species, which is compatible with either a slow monomer–dimer equilibrium or the existence of both phosphorylated and unphosphorylated BasR species due to substoichiometric phosphorylation. Nevertheless, FP–BasR could dimerize upon phosphorylation despite the absence of observable FRET. Taken together, phosphorylation-promoted multimerization appears to be a common feature for all *E. coli* OmpR/PhoB subfamily RRs. Such interactions do not require the binding of the RR to DNA, although DNA binding might be expected to enhance the interaction.

### Limitations of *in vitro* analysis

As suggested in FP–BasR interactions, FRET intensities can be restricted by levels of RR phosphorylation *in vitro*. Some full-length RRs have been shown to be substoichiometrically phosphorylated by phosphoramidate *in vitro*, attributable in at least one case to decreased phosphotransfer rates resulting from inhibitory interactions of the N-terminal receiver domain and the C-terminal DNA-binding domain (Friedland *et al*., 2007). Slightly different FRET profiles were observed for full-length CpxR and the N-terminal receiver domain of CpxR, potentially resulting from either different phosphorylation levels and/or direct interactions of the DNA-binding domains. Future investigation of FRET profiles of all the N-terminal domains of *E. coli* OmpR/PhoB subfamily RRs will be valuable to address the contributions of the C-terminal output domain. An additional consideration is that RR phosphorylation by small-molecule phosphodonors is generally less efficient than phosphorylation by the cognate HK (Silversmith *et al*., 1997; Da Re *et al*., 1999b; Mayover *et al*., 1999). For some RRs in our experiments with phosphoramidate as phosphodonor, phosphorylation did not reach a steady state even after 55 min. A faster rate of phosphotransfer from the cognate HK with a comparable rate of RR dephosphorylation would achieve both more rapid kinetics and a greater steady-state stoichiometry of phosphorylation. Thus the phosphorylation-induced interaction observed in our FRET studies might be expected to under-represent interactions that might be promoted by a higher level of RR phosphorylation catalysed by an activated HK *in vivo*. Understanding the regulatory roles of homodimers and heterodimers will require the characterization of both RR interaction and phosphorylation *in vivo*.

Our systematic FRET measurements do not provide details of the interaction for all RRs, for instance, the oligomeric states or the exact *K*_D_ values, as it is not practical to determine the concentration-dependent FRET for all possible RR pairs. FRET between FP-fused RRs could result from RR dimerization as well as interactions of RR dimers or formation of higher-order oligomers. Extensive analyses of FP–PhoB do suggest dimer formation and give a *K*_D_ value comparable to that determined by other methods. Moreover, the distance-dependent FRET parameters can also be deduced from the in-depth FRET measurement of FP–PhoB. Assuming the same positioning of fluorescent proteins for all RR pairs, the FRET ratio changes can potentially be used to estimate the *K*_D_ based on the FRET parameters determined from the FP–PhoB dimerization model. However, many homo-pairs, such as TorR, CusR and ArcA, showed an exceptionally large increase of the FRET ratio, giving unrealistic *K*_D_ values (data not shown). Hence the original assumption of identical positioning of FP fluorophores seems to be incorrect or these RRs can form higher-order oligomers leaving the dimerization model unsuitable for calculations. Indeed ArcA has been shown to form oligomers in solution and TorR oligomerization has been suggested to play a role in gene regulation (Simon *et al*., 1995; Jeon *et al*., 2001; Toro-Roman *et al*., 2005a). The exact structural details of oligomerization are not known, but as the structures of the receiver domains of TorR and ArcA show an α4-β5-α5 dimerization interface similar to that of other OmpR/PhoB subfamily RRs (KdpE, PhoB and PhoP) (Bachhawat *et al*., 2005; Toro-Roman *et al*., 2005a,b; Bachhawat and Stock, 2007), higher-order oligomers are assumed to involve associations of this dimeric unit. It is very common that multiple RR binding sites are located upstream of a promoter and the cooperative binding of multiple RR dimers is important to regulation (Harlocker *et al*., 1995; Simon *et al*., 1995), as seen in the regulation of *ompC* and *ompF* by OmpR (Yoshida *et al*., 2006).

### Specificity of OmpR/PhoB RR dimerization

The sequences in the α4-β5-α5 region are highly conserved within the subfamily, a characteristic that distinguishes the OmpR/PhoB subfamily from other RRs, and many conserved residues come from residues involved in formation of important intermolecular contacts. All these features of sequences, structures and protein–protein interactions are consistent with a model of phosphorylation promoting the formation of a common active dimer state within the OmpR/PhoB subfamily. Even though a conserved dimer structure is suggested for subfamily members, their interactions still remain specific. Homo-pairs of FP–RRs gave the strongest FRET while meaningful FRET signals between hetero-pairs were restricted to a small number of RRs. It seems that RRs prefer to interact with themselves and the interactions of hetero-pairs are weak compared with homo-pairs.

The specificity likely arises from interacting side-chains in the α4-β5-α5 interface because the backbones of α4-β5-α5 regions are well aligned in five available active dimer structures (ArcA, KdpE, PhoB, PhoP and TorR) and are thus not expected to contribute significantly to the interaction specificity. A distinctive feature for the α4-β5-α5 dimerization interface is the formation of an extensive network of salt bridges by a conserved set of charged residues (Toro-Roman *et al*., 2005a). Two charged side-chains (*d* positions in Fig. 1C) are absolutely conserved at the centre of the dimerization interface and have been proved crucial for the dimerization of PhoB (Mack, 2008). Other salt-bridge-forming residues are usually conserved but not present in all the OmpR/PhoB subfamily members of *E. coli*, such as the residues corresponding to E94 and R115 in ArcA (*b* positions in Fig. 1C). These two positions are occupied by a reverse pair of charged residues in QseB and two polar residues in PhoP. Interestingly, both QseB and PhoP did not show any intermediate level of FRET with other RRs thus the unique residues at these two positions may play a role in their interaction specificity. A few other positions display similar patterns of variance with only one or two RRs deviating from the consensus and there is one peripheral contacting pair with slightly higher variations. Cumulatively, the combination of these subtle changes at different positions, as well as potential conformational perturbations of the conserved interface residues from distant parts of the structure, may contribute to the interaction specificity within the OmpR/PhoB subfamily.

A proper and accurate response to stimuli relies heavily on the specificity of signalling components to keep the signalling pathways insulated from one another. The mechanisms for signalling specificity have attracted great interest in many different systems (Pawson and Nash, 2000; Laub and Goulian, 2007; Ubersax and Ferrell, 2007). One of the central issues for the specificity involves correct molecular recognition along the pathway. The two-component system usually features a series of recognition events, including the dimerization of HKs for autophosphorylation, the HK–RR interaction for phosphotransfer and, for most RRs, the dimerization of RRs and DNA recognition for transcriptional control. It has been shown that HKs exhibit a global kinetic preference for phosphotransfer to their cognate RRs (Skerker *et al*., 2005), which is likely mediated by the specific interaction between a HK and its cognate RR (Skerker *et al.*, 2008). Here we demonstrate that RR dimerization is also specific with little cross-talk between highly similar proteins. All these precise recognition events ensure that one stimulus does not mistakenly activate another pathway, thus avoiding the commitment of resources to futile activities.

### Heterodimerization of OmpR/PhoB RRs

On the other hand, the homodimerization specificity does not completely exclude the possibility for heterodimer formation. FRET signals between hetero-pairs were observed for a small number of RRs. Some of these interactions are between RR pairs with highly similar sequences, e.g. YedW and CusR. CusR belongs to the copper-responsive CusS/CusR two-component system (Munson *et al*., 2000) and shares ∼50% sequence identity with YedW. YedV, the cognate HK of YedW, has been shown to transphosphorylate CusR *in vitro* although it is not clear whether such cross-talk would occur *in vivo* (Yamamoto *et al*., 2005). Nevertheless these two systems are closely related as copper can also activate the transcription of *yedVW* in a CusR-dependent manner (Yamamoto and Ishihama, 2006). The interaction between CusR and YedW might result from the sequence similarity and provide potential regulatory roles. Similar examples are the interactions between YFP–PhoB and CFP–BaeR or CFP–KdpE. A few non-cognate HKs, including CreC, KdpD, BaeS and more, have been found to activate PhoB in the absence of the cognate HK PhoR and cross-regulation has been implied among the PhoBR, CreBC and BaeRS systems, although cross-regulation is likely at the transcription level instead of the level of transphosphorylation (Amemura *et al*., 1990; Wanner, 1992; Nishino *et al*., 2005; Zhou *et al*., 2005; Laub and Goulian, 2007). It is possible that these interactions between RR hetero-pairs might be involved in cross-regulation.

Hetero-pair interactions are not restricted to RR pairs with close sequence similarity but also include more distant pairs, such as KdpE/RstA and CusR/CpxR. It is especially interesting that CpxR is able to interact with multiple RRs, such as ArcA, PhoB, OmpR, BaeR, CusR and more. The CpxAR two-component system is a global regulatory system that responds to cell envelope stress (De Wulf *et al*., 2002; Ruiz and Silhavy, 2005; Dorel *et al*., 2006). Cell envelope integrity and cell surface properties are obviously closely monitored by bacterial cells and can influence a wide variety of regulatory pathways. For instance, the copper stress response and oxygen limitation response are tightly correlated with the CpxR-mediated envelope stress response (Yamamoto and Ishihama, 2006; Partridge *et al*., 2007). It has been shown that CpxR also regulates genes that are controlled by other RRs, such as the redox regulator ArcA (Partridge *et al*., 2007), the osmoregulator OmpR (Batchelor *et al*., 2005; Jubelin *et al*., 2005) and another envelope stress regulator BaeR (Raffa and Raivio, 2002; Hirakawa *et al*., 2005), with some even having overlapping DNA binding sites. The interactions between these hetero-pairs could certainly play a role in cross-regulation of CpxR with these different RRs.

Traditionally, transcription co-regulation by different RRs is presumed to occur through simultaneous binding of different RR homodimers to the same promoter for concerted regulation. The formation of RR heterodimers gives rise to a distinct co-regulation mechanism in which heterodimers can influence or compete with the formation of homodimers. However, the hetero-pair interactions are generally weaker than the interaction of homo-pairs as indicated by the FRET signal intensity. Thus, there might not be a significant amount of heterodimers available when the level of phosphorylated RR is low. A few RRs, such as PhoB, CusR and CpxR, can activate their own expression (Guan *et al*., 1983; Raivio *et al*., 1999; Hoffer *et al*., 2001; Yamamoto and Ishihama, 2005), giving higher concentration of phosphorylated RR when the system is fully activated, which could potentially allow heterodimer formation even with a low affinity. Moreover, heterodimers can align two different DNA-binding domains, providing the potential for recognition of a unique set of hybrid sites with each of the two half-sites corresponding to the recognition elements of different RRs. As DNA binding to tandem sites has been shown to enhance RR homodimerization (Maris *et al*., 2005), binding to such heterodimer-specific DNA sites might also increase the heterodimerization affinity to allow sufficient heterodimers to form. The potential regulatory roles of heterodimers can be either negative or positive as non-functional heterodimers can serve as dead-end complexes to compete with homodimers, while functional heterodimers can recognize novel sites to regulate transcription of a completely different set of genes from those regulated by either parent homodimer. Such regulation has not yet been reported in OmpR/PhoB subfamily RRs, but precedent exists in heterodimers of the LytR subfamily RRs, ComE and BlpR, which have been suggested to recognize a hybrid DNA motif for convergent gene regulation (Knutsen *et al*., 2004).

In summary, our FRET analyses provide a systematic view of RR interactions in two-component signal transduction pathways of *E. coli* and demonstrate the interaction specificity within the largest RR subfamily, the OmpR/PhoB subfamily. It is not clear whether such specificity is evolutionarily optimized within individual organisms and whether cross-reactions could occur more readily between paralogous proteins from different species as shown in interactions of Src homology 3 (SH3) domains (Zarrinpar *et al*., 2003). Specific RR interactions, together with phosphotransfer specificity, ensure signal transmission fidelity for individual HK–RR pathways despite the highly conserved core structures and functions shared by a large number of different two-component proteins in a cell. However, the specificity between signalling pathways does not mean the isolation of individual pathways. Instead, cells co-ordinate responses to a complex environment through a network of interacting pathways. It is well known that the connections and communications of signalling proteins greatly contribute to the complexity of eukaryotic signalling networks (Bhattacharyya *et al*., 2006). Recently it has begun to emerge that two-component signal transduction is not merely comprised of linear pathways but that cross-regulation does occur at multiple levels (see reviews by Bijlsma and Groisman, 2003; Laub and Goulian, 2007). One of the common mechanisms of signal integration is through converging distinct pathways to the transcription regulation of overlapping groups of genes. Our discovery of interactions between RR hetero-pairs raises the possibility of an additional mechanism that could facilitate cross–regulation. These interactions may play a significant role in integrating and co-ordinating responses to a wide variety of environmental stimuli.

## Experimental procedures

### Strains and plasmids

The strains and plasmids used in this study are listed in Table S1. Two mutations (A206K/Q69K) were introduced into EYFP (Clontech) gene to create monomeric mYFP. mYFP and ECFP (Clontech) were fused with a long flanking promoter sequence from pET21b at the 5′ end and a linker sequence encoding GGGGGHM at the 3′ end by recombinant PCR. Subsequently they were inserted into the T7 polymerase-based vector pET21b (Novagen) between the SphI and NdeI sites to give pRG85 and pRG31. The plasmid pRG85 was then used as a template to amplify mYFP with a trypsin-susceptible linker sequence encoding GGGLVPRGSGGHM added at the 3′ end and the PCR fragment was ligated back into pET21b with SphI and NdeI to yield pRG88. Plasmids pRG31 and pRG88 were used for all the subsequent cloning of FP–RR fusions. RR genes were amplified from *E. coli* DH5α or BW25113 genomic DNA by PCR and inserted into either pRG31 or pRG88 at the NdeI and HindIII sites to create the CFP–RR and YFP–RR series. All of the above plasmids were confirmed by sequencing and used for protein expression. DNA fragments encoding PhoB and CpxR were also inserted into pET21b at the NdeI and HindIII sites to create expression plasmids for His_6_-tagged PhoB and CpxR. Similarly, the coding sequence for ArcA was amplified with a Flag tag at the C-terminus and ligated into pET21b. To achieve IPTG-regulated low-level expression of FP–PhoB for *in vivo* activity assays, a low-copy-number plasmid with a *lac* promoter, pRG2, was created by three-piece ligation of the EcoRI/HindIII fragment from pTRM11 (encoding PhoB), the XmnI/HindIII-digested pET21b (containing a NotI site) and the EcoICRI/EcoRI-digested pMLB1120.215 (a low-copy expression vector). CFP–PhoB and YFP–PhoB fragments were then excised from corresponding plasmids by XbaI and NotI digestion and ligated into pRG2 to give pRG20 and pRG94.

### Protein expression and purification

The plasmids encoding FP–RRs were transformed into *E. coli* strain BL21(DE3) (Novagen). Cells were grown at 37°C in Luria–Bertani (LB) medium supplemented with 100 μg ml^−1^ ampicillin. After reaching mid-exponential phase, the cultures were cooled and induced by 0.5 mM IPTG at 20°C overnight. All of the following purification steps were performed at 4°C. Cells were harvested by centrifugation for 20 min at 4000 *g* and stored at −80°C until needed. To purify the His_6_-tagged FP–RR proteins, cell pellets were re-suspended in binding buffer [20 mM sodium phosphate, 0.5 M NaCl, 20 mM imidazole, 2 mM 2-mercaptoethanol (β-ME), pH 7.4] and lysed by sonication followed by clarification at 18000 *g* for 30 min. The clarified lysates were loaded onto two tandem 1 ml HisTrap FF affinity columns (GE Healthcare), washed with 50 ml of binding buffer and eluted with a 40 ml gradient of 20–500 mM imidazole in binding buffer. Fluorescent fractions containing FP–RR proteins were pooled and dialysed against the reaction buffer (50 mM Tris-HCl, 100 mM NaCl, 2 mM β-ME, pH 7.4). To prepare FP–PhoB samples for SV analyses, FP–PhoB proteins were initially purified using the HisTrap FF affinity columns with a similar protocol as above. Then the eluants were pooled and a 4 M (NH_4_)_2_SO_4_ solution was slowly added to the solution to reach 1 M. The samples were then loaded onto a HiLoad phenyl Sepharose 16/10 column (Amersham Biosciences) and eluted with a gradient of 1.0 M to 0 M (NH_4_)_2_SO_4_ in a buffer of 20 mM Tris-HCl and 2 mM β-ME, pH 7.8. Fractions containing FP–PhoB proteins were finally purified by gel filtration chromatography using a Superdex75 26/60 column (Amersham Biosciences). All the FP–RR proteins were filtered through 0.2 μm filters before subsequent experiments and the concentrations were determined by absorbance at 433 nm for CFP–RRs and at 514 nm for YFP–RRs using respective molar extinction coefficients (CFP, 32 500 M^−1^ cm^−1^; YFP, 83 400 M^−1^ cm^−1^) (Shaner *et al*., 2005).

His_6_-tagged PhoB and CpxR proteins were expressed similarly as FP fusion proteins. Cell lysates were clarified by ultracentrifugation for 60 min at 80 000 *g* and loaded onto a 5 ml HisTrap column (GE Healthcare). Unbound proteins were washed off with 100 ml of binding buffer and His_6_-tagged proteins were eluted with a 100 ml gradient of 30–500 mM imidazole. Fractions containing His_6_-tagged proteins were pooled, and subjected to gel filtration chromatography using a Superdex75 26/60 column equilibrated with 20 mM Tris-HCl, 100 mM NaCl, 2 mM β-ME, pH 7.4. Lysates from cells overexpressing the Flag-tagged ArcA were clarified by ultracentrifugation for 60 min at 80 000 *g*. A saturated solution of (NH_4_)_2_SO_4_ was added to the supernatant to 60% (w/v) and the resulting protein pellet was re-suspended and dialysed in buffer A (20 mM Tris-HCl and 2 mM β-ME, pH 7.5). The dialysed sample was loaded onto two tandem 5 ml HiTrap Q columns (Amersham Biosciences) and eluted with a 200 ml gradient of 0–1.0 M NaCl in buffer A. Fractions containing Flag-tagged ArcA were further purified with a HiLoad phenyl Sepharose 16/10 column and finally a Superdex75 26/60 gel filtration column as described earlier (Toro-Roman *et al*., 2005a).

### Assay of AP activity

Plasmids pRG20 and pRG94 were transformed into *phoB* deletion strain JWK0389-1 (Baba *et al*., 2006). Cells from overnight LB cultures were inoculated into MOPS minimal media containing 5 mM (high phosphate) or 0.1 mM (low phosphate) KH_2_PO_4_ and grown for 4–5 h. To ensure similar protein expression levels of FP–PhoB and wild-type PhoB, different concentrations of IPTG were used to induce the expression and protein levels were analysed by Western blot using anti-PhoB antibody. IPTG induction at 50 μM gave comparable protein levels of CFP–PhoB, YFP–PhoB and wild-type PhoB under the low-phosphate condition therefore 50 μM IPTG was included in the MOPS minimal media. Similar amounts of bacterial cells (∼0.3 OD ml) were harvested based on the optical density (OD) measured at 595 nm. Subsequently, the cells were re-suspended in 0.5 ml of 0.5 M Tris-HCl, pH 8.0; 40 μl of CHCl_3_ and 40 μl of 0.1% SDS were then added to each sample followed by vigorous shaking and clarification at 13000 *g*. One hundred microlitres of supernatant from every sample was transferred to a 96-well plate and the reaction was initiated by addition of 100 μl of 4 mM *p*-nitrophenylphosphate. The absorbance at 420 nm was followed continuously and the rate of absorbance increase was calculated to represent the AP activity. All AP activities were normalized to the AP activity of wild-type cells under low-phosphate conditions.

### SV analysis

Sedimentation velocity experiments were performed with an Optima XL-I analytical ultracentrifuge (Beckman) using absorbance optics. Proteins were diluted to the indicated concentrations in the reaction buffer containing 5 mM MgSO_4_, and 40 mM phosphoramidate was added if phosphorylation was desired. Epon double-sector centrepieces with quartz windows were filled with protein samples and the reaction buffer respectively. The samples were centrifuged at 45 000 r.p.m. using an An-60 Ti rotor at a temperature of 4°C. The buffer density, viscosity and partial specific volumes of individual proteins were calculated using the program sednterp (Laue *et al*., 1992). SV data were analysed with the program sedfit to generate the *c*(*s*) distribution (Schuck, 2000).

### Fluorescence analyses

Fluorescence was measured by a FluoroMax-3 fluorometer (HORIBA Jobin Yvon) at 25°C. Fluorescence emission spectra for CFP–RRs were scanned from 460 nm to 560 nm with 433 nm excitation and YFP emission spectra were scanned from 500 nm to 580 nm with excitation at 488 nm. For tryptophan fluorescence of PhoB, the emission intensity was monitored at 345 nm with excitation at 295 nm. For FRET measurements, emissions at 475 nm and 527 nm were recorded with excitation at 433 nm and the FRET ratio was defined as the ratio of 527:475 nm emissions. CFP–RR and YFP–RR were diluted to the indicated concentrations in the reaction buffer of 50 mM Tris-HCl, 100 mM NaCl, 2 mM β-ME, pH 7.4 and phosphoramidate was added to 20 mM if phosphorylation was desired. To initiate the phosphorylation, 5 μl of 1 M MgSO_4_ was added to 1 ml of the sample in the cuvette. The FRET ratio before phosphorylation was subtracted to calculate the change of FRET ratio. For the PhoB competition experiment, after the FRET ratio reached a plateau for the phosphorylated CFP–PhoB/YFP–PhoB mixture, 20 μl of PhoB (68 μM) was titrated into 1 ml of mixture. Additions were spaced by 10 min intervals to allow phosphorylation of newly added PhoB and the tryptophan fluorescence quenching also indicated that the phosphorylation reached a steady state before the next addition. Similar protocols were followed for the titration of ArcA into theCFP–ArcA/YFP–CpxR mixture. To examine the effect of DNA binding on FP–OmpR interaction, two complementary oligonucleotides were heated to 85°C and slowly cooled to room temperature to generate the DNA duplex corresponding to the C1 DNA binding site from the *ompC* promoter (5′-TCCCTTGCATTTACATTTTGAAACATCTATAGCGAT-3′). Five microlitres of C1 DNA (77 μM) was repeatedly titrated into 2 ml of the FP–OmpR mix and the fluorescence was measured.

To determine the *K*_D_ of FP–PhoB, CFP fluorescence at 475 nm was measured for CFP–/YFP–PhoB mixtures at different concentrations. YFP–PhoB (30 μM) and indicated concentrations of CFP–PhoB were mixed and phosphorylated prior to the titration of 20 μl of the mixture into 1 ml of phosphorylated CFP–PhoB at the same indicated concentrations. Experiments were performed at three different initial CFP–PhoB concentrations and the fluorescence values were recorded. The decreases of CFP fluorescence were plotted against the concentration of YFP–PhoB and simultaneous fitting of all three curves was performed using OriginPro8 (OriginLab).

### Systematic analyses of FRET between FP–RR pairs

FRET between all the FP–RR pairs were determined with a Varioskan fluorescence plate reader (Thermo Electron Corporation). Fluorescence was measured at 475 nm and 527 nm with excitation at 430 nm at 30°C. CFP–RR and YFP–RR were diluted into the reaction buffer and the total volume was 140 μl. Phosphorylation was initiated by addition of 40 μl of phosphoramidate and MgSO_4_. The final concentrations were 0.6 μM CFP–RR, 2.5 μM YFP–RR, 20 mM phosphoramidate and 5 mM MgSO_4_. Fluorescence was followed immediately after the addition and every 450 s thereafter until 3150 s. FRET ratio changes were calculated as described earlier.

### Equilibrium calculations

There are four potential equilibria coexisting for a given hetero-pair:
(1)
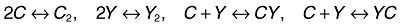

because the *CY* and *YC* heterodimers may have different dissociation constants due to the asymmetric tandem arrangement of their DNA-binding domains. The dissociation constants are given by
(2)
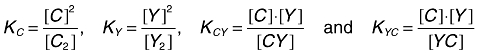

The conservation of mass gives
(3)
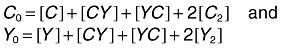

where *C*_0_ and *Y*_0_ are the initial concentrations of CFP–RR and YFP–RR. Consider a homo-pair such as CFP–PhoB and YFP–PhoB, the dissociation constants are equal:
(4)
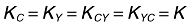

The concentration of FRET-capable dimers (*CY* and *YC*) is the sum of [*CY*] and [*YC*], which can be derived from solving Eqns 2, 3 and 4:
(5)
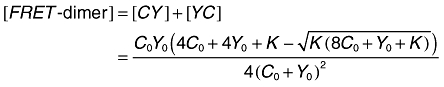

and the fluorescence change is proportional to the concentration of FRET-capable dimers:
(6)
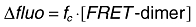

where *f*_*c*_ is a distance-dependent parameter correlated with the FRET efficiency. Initial concentrations of CFP–/YFP–PhoB (*C*_0_ and *Y*_0_) are known and fluorescence changes can be measured thus the dissociation constant *K* and the FRET parameter *f*_*c*_ can be estimated from the fitting of fluorescence measurements at multiple concentration series with Eqns 5 and 6.
