# Supplementary material for: System-level mapping of Escherichia coli response regulator dimerization with FRET hybrids
Source: Mol Microbiol. 2008 Jul 15;69(6):1358–72. doi: 10.1111/j.1365-2958.2008.06355.x (PMC2586830; doi:10.1111/j.1365-2958.2008.06355.x)
Supplement: Supplementary file 1 [file mmi0069-1358-SD1.pdf]

Supplementary Material

Table S1. Strains and Plasmids

Strains / plasmids	Relevant characteristics	Reference / source
Strains		
E. coli		
DH5α	General cloning strain	Invitrogen
BL21(DE3)	F^-, *omp*T, *hsd*S_B(r_B-, m_B-), *dcm*, *gal*, λ(DE3), expression strain	Novagen
BW25113	*lacI* ^{q} *rrnB*$_{T14}$ Δ*lacZ*$_{WJ16}$ *hsdR514* Δ*araBA-D*$_{AH33}$ Δ*rhaBAD*$_{LD78}$, K-12 derivative	(1)
JWK0389-1	Δ*phoB*, BW25113 derivative	(2)
Plasmids		
pET21b	T7 polymerase-based expression vector, Ap^r	Novagen
pRG31	vector for construction of *cfp*-RR fusions, pET21b derivative, Ap^r	This study
pRG85	pET21b derivative, Ap^r	This study
pRG88	vector for construction of *yfp*-RR fusions, pET21b derivative, Ap^r	This study
pRU850	*cfp-arcA*, pET21b derivative, Ap^r	This study
pJZG130	*yfp-arcA*, pRG88 derivative, Ap^r	This study
pRG51	*cfp-torR*, pRG31 derivative, Ap^r	This study
pRG93	*yfp-torR*, pRG88 derivative, Ap^r	This study
pRG53	*cfp-ompR*, pRG31 derivative, Ap^r	This study
pRG91	*yfp-ompR*, pRG88 derivative, Ap^r	This study
pRU853	*cfp-cpxR*, pET21b derivative, Ap^r	This study
pRG122	*yfp-cpxR*, pRG88 derivative, Ap^r	This study
pRG153	*cfp-rstA*, pRG31 derivative, Ap^r	This study
pRG156	*yfp-rstA*, pRG88 derivative, Ap^r	This study
pRU856	*cfp-phoB*, pET21b derivative, Ap^r	This study
pRG90	*yfp-phoB*, pRG88 derivative, Ap^r	This study
pRG130	*cfp-baeR*, pRG31 derivative, Ap^r	This study
pRG138	*yfp-baeR*, pRG88 derivative, Ap^r	This study
pRG135	*cfp-creB*, pRG31 derivative, Ap^r	This study
pRG143	*yfp-creB*, pRG88 derivative, Ap^r	This study
pRG50	*cfp-kdpE*, pRG31 derivative, Ap^r	This study
pRG92	*yfp-kdpE*, pRG88 derivative, Ap^r	This study
pRG52	*cfp-phoP*, pRG31 derivative, Ap^r	This study
pJZG129	*yfp-phoP*, pRG88 derivative, Ap^r	This study
pRG133	*cfp-cusR*, pRG31 derivative, Ap^r	This study
pRG141	*yfp-cusR*, pRG88 derivative, Ap^r	This study
pRG155	*cfp-yedW*, pRG31 derivative, Ap^r	This study
pRG157	*yfp-yedW*, pRG88 derivative, Ap^r	This study
pRG132	*cfp-basR*, pRG31 derivative, Ap^r	This study
pRG140	*yfp-basR*, pRG88 derivative, Ap^r	This study
pRG131	*cfp-qseB*, pRG31 derivative, Ap^r	This study
pRG139	*yfp-qseB*, pRG88 derivative, Ap^r	This study
pRG184	*cfp-fixJ*, pRG31 derivative, Ap^r	This study
pRG183	*yfp-fixJ*, pRG88 derivative, Ap^r	This study
pRG129	*cfp-ntrC*n, pRG31 derivative, Ap^r	This study
pRG137	*yfp-ntrC*n, pRG88 derivative, Ap^r	This study
pJZG132	*cfp-narL*, pRG31 derivative, Ap^r	This study
pJZG131	*yfp-narL*, pRG88 derivative, Ap^r	This study
pJZG53	*phoB-His*$_6$, pET21b derivative, Ap^r	This study
pJZG54	*cpxR-His*$_6$, pET21b derivative, Ap^r	This study
pJZG55	*arcA-flag*, pET21b derivative, Ap^r	This study
pMLB1120.215	*lac* promoter, low copy number, Ap^r	(3)
pTRM11	P_{lac}-*phoB*, pMLB1120.215 derivative, Ap^r	(4)
pRG2	*lac* promoter, pMLB1120.215 derivative, Ap^r	This study
pRG20	P_{lac}-*cfp-phoB*, pMLB1120.215 derivative, Ap^r	This study
pRG94	P_{lac}-*yfp-phoB*, pMLB1120.215 derivative, Ap^r	This study

(1) Datsenko, K. A. and Wanner, B. L. 2000. *Proc. Natl. Acad. Sci. USA* 97: 6640.
(2) Baba, T., Ara, T. *et al*. 2006. *Mol. Syst. Biol.* 2: Epub.
(3) Obtained from M. Berman, Litton Institute. of Applied Biotechnology, Rockville, MD
(4) Mack, T. 2008. Ph.D. thesis, University of Medicine and Dentistry of New Jersey.

Fig. S1. Cleavage of YFP-PhoB by trypsin. YFP-PhoB (2.5 μM) was incubated with 5 μg/ml trypsin for the indicated time at room temperature. The digestion was stopped by adding 4xSDS gel loading buffer prior to PAGE and staining with Coomassie blue. YFP-PhoB ran at ~55 kDa and trypsin digestion resulted in disappearance of the 55 kDa band and emergence of lower M.W. bands corresponding to PhoB and YFP. The cleavage appeared complete at 15 min and the intensity of the cleaved YFP band did not decrease after 30 min of digestion, suggesting that YFP is relatively stable at the experimental trypsin concentration.

Fig. S2. *c*(*s*) distribution of FP-PhoB (A) and YFP-BasR (B)

A. *c*(*s*) distribution of the mixture of 5.5 μM CFP-PhoB and 3.0 μM YFP-PhoB. The distribution is from the same SV experiment described in Fig. 3E. SV profiles were collected at both the CFP absorbance 433 nm and YFP absorbance 514 nm. The CFP profiles (dark cyan) are similar to the YFP profiles (orange); both feature a major peak at 3.7 S for unphosphorylated samples (solid lines) and a shifted peak at 5.4 S for phosphorylated samples (dotted lines), consistent with dimerization of both CFP-PhoB and YFP-PhoB once phosphorylated.

B. *c*(*s*) distribution of YFP-BasR. SV profiles were collected for YFP-BasR at a loading concentration of 6.9 μM with the absorbance optics at 514 nm. The unphosphorylated sample displayed a major peak at 3.6 S, similar to unphosphorylated FP-PhoB proteins that have a similar M.W. of 55 kDa. The phosphorylated sample showed two major peaks. One is at 5.4 S, a similar sedimentation coefficient as phosphorylated FP-PhoB, suggesting the dimerization of YFP-BasR; the other is at 3.6 S, the same as the unphosphorylated samples. The presence of the 3.6 S peak indicates a monomer species that is not in fast equilibrium with the dimer. This could result from incomplete phosphorylation or a slow dimerization and low affinity for YFP-BasR,

which might also account for the absence of any significant FRET observed for FP-BasR homo-pairs.

Fig. S3. Systematic analyses of FRET between FP-RRs. The data are from the same experiment described in Fig. 6 with more details shown here.

A. FRET of RR homo-pairs. RRs included here are all the remaining homo-pairs not shown in Fig. 6A. Phosphorylation increased FRET for all RR homo-pairs except NarL and the receiver domain of NtrC (NtrCn).

B. Phosphorylation-dependent FRET of RR homo-pairs. The change of FRET ratio at the end of the experiment (time 3150 s) was used to evaluate FRET signal of phosphorylated (grey) and unphosphorylated (white) pairs. Both FP-TorR and FP-RstA pairs showed FRET even without phosphorylation, but phosphorylation further increased the FRET ratio.

C. Standard deviations of FRET ratio changes for phosphorylated FP-RR pairs. The data are from four independent experiments. Nearly all the FP-RR pairs have rather small standard deviations. The three highest ones are CFP-YedW/YFP-YedW, CFP-RstA/YFP-RstA and CFP-RstA/YFP-KdpE. The reason for the high variation is not clear and could be due to protein stability. But the FRET ratio changes of these three pairs are still considerably higher than the background even with high standard deviations.

D. FRET of non-phosphorylated FP-RR pairs. Experimental methods and conditions were similar to those of phosphorylated samples except for the absence of phosphoramidate in the reaction mixtures.

Fig. S4. Phosphorylation-dependent CFP-CpxR Emission Spectra and FRET between CFP-CpxR and Other YFP-RRs. All emission spectra were measured with a fluorometer at

25°C and the samples were in 50 mM Tris-HCl, 0.1 M NaCl and 2 mM β-ME, pH 7.4. FRET between CFP-CpxR and other YFP-RRs were measured by a fluorescence platereader.

A. Emission spectra of CFP-ArcA with the excitation at 433 nm. CFP-ArcA (1 μM) displays a typical CFP emission spectrum with the highest peak at 475 nm (black solid line). Addition of 2.4 μM CpxR (red solid line) or phosphorylation by 20 mM phosphoramidate (green dotted line) does not change the spectrum. When fluorescence is normalized to the peak fluorescence at 475 nm, these spectra look identical to each other.

B. Emission spectra of CFP-CpxR with the excitation at 433 nm. The spectrum of CFP-CpxR is different from a typical CFP emission spectrum. The peak shifts from 475 nm to 490 nm (black solid line). The reason for the spectrum change is not clear and it may originate from the interaction of CpxR with CFP. But addition of CpxR to CFP-ArcA did not alter the CFP emission (Fig. S4A), thus the hypothesized CpxR/CFP interaction is only restricted to the linked proteins and appears to be negligible at experimental concentrations for free CpxR and CFP-RRs. The spectrum change is specific to CFP-CpxR as YFP-CpxR shows identical emission spectra as other YFP-RRs (data not shown). Further, phosphorylation alters the emission spectrum of CFP-CpxR (green dotted line), resulting in an increase of emission at 475 nm and not much change at 527 nm. Therefore, phosphorylation decreases the yellow to cyan fluorescence ratio.

C and D. FRET between CFP-CpxR and other YFP-RRs. The time-courses of FRET ratio change upon phosphorylation are shown in (C) and the FRET ratio changes at the last time point are compared in (D). For most pairs, phosphorylation decreases the FRET ratio. Since it is unlikely that CFP-CpxR interacts with free YFP in solution at the experimental concentrations, the FRET ratio change of CFP-CpxR/YFP pair (dotted line in D) is considered to be the intrinsic change of FRET ratio due to CFP-CpxR phosphorylation. Any FRET ratio change not significantly deviating from that of CFP-CpxR/YFP is considered to be lacking any significant

FRET while the values above the dotted line suggest potential interactions. To correct for this effect, the absolute value of FRET ratio change of the CFP-CpxR/YFP pair was added to the FRET ratio change for all the CFP-CpxR/YFP-RR pairs to give positive values of FRET ratio changes as shown in Fig. 6.

Figure S1

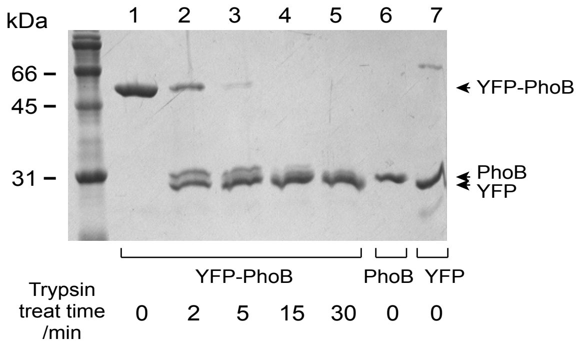
kDa
1
2
3
4
5
6
7
66
45
31
YFP-PhoB
PhoB
YFP
YFP-PhoB
PhoB
YFP
Trypsin treat time /min
0
2
5
15
30
0
0

Figure S2

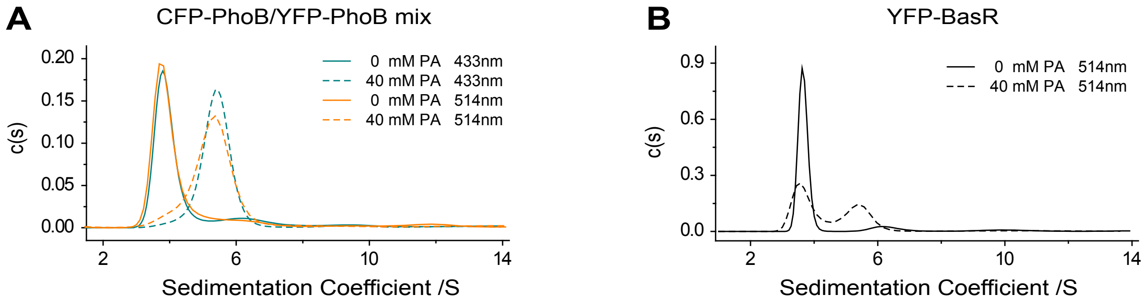
A
CFP-PhoB/YFP-PhoB mix
0 mM PA 433nm
40 mM PA 433nm
0 mM PA 514nm
40 mM PA 514nm
c(s)
0.20
0.15
0.10
0.05
0.00
2
6
10
14
Sedimentation Coefficient /S
B
YFP-BasR
0 mM PA 514nm
40 mM PA 514nm
c(s)
0.9
0.6
0.3
0.0
2
6
10
14
Sedimentation Coefficient /S

Figure S3

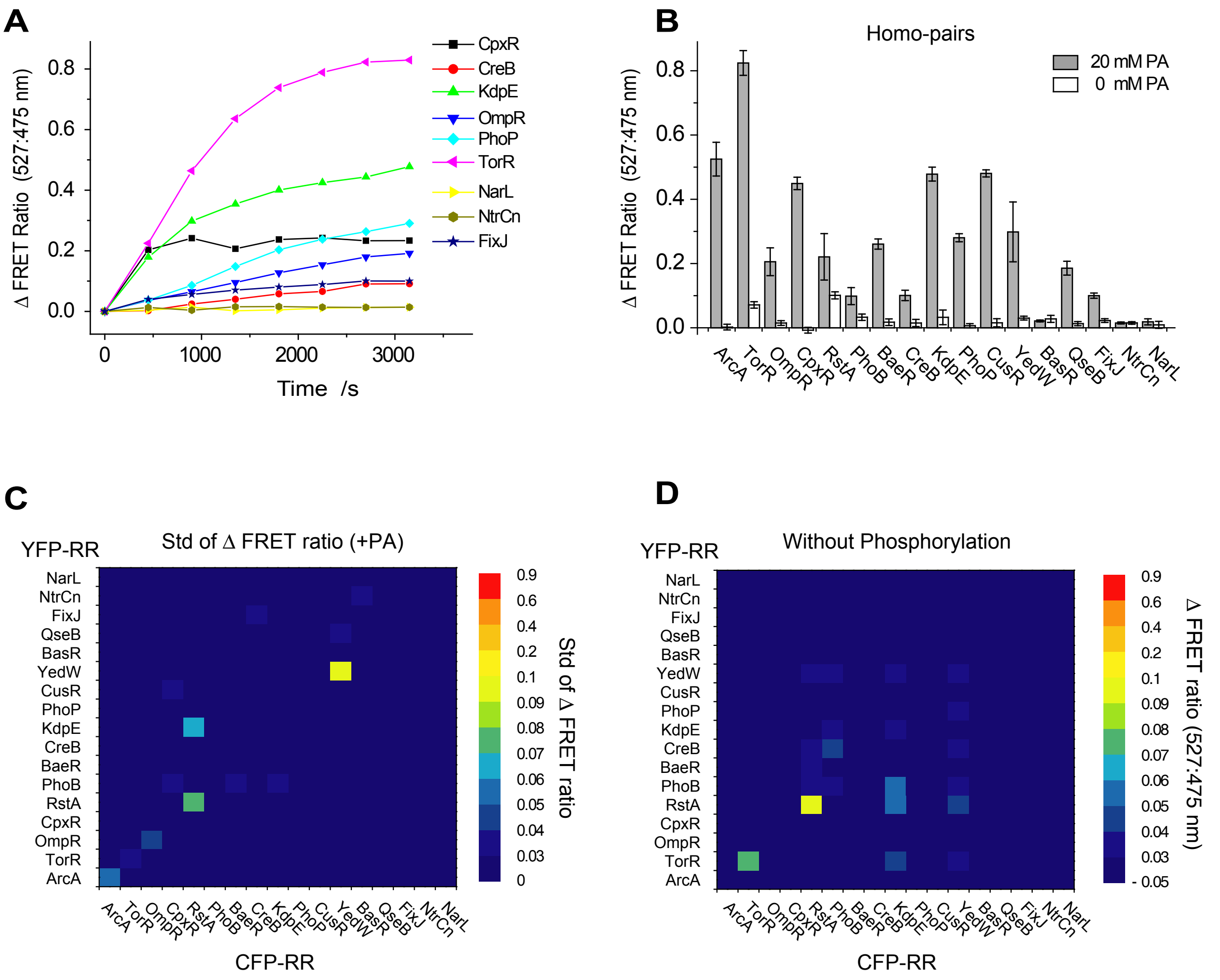
A
Δ FRET Ratio (527:475 nm)
0.0
0.2
0.4
0.6
0.8
0
1000
2000
3000
Time /s
CpxR
CreB
KdpE
OmpR
PhoP
TorR
NarL
NtrCn
FixJ
B
Homo-pairs
Δ FRET Ratio (527:475 nm)
20 mM PA
0 mM PA
ArcA
TorR
OmpR
CpxR
RstA
PhoB
BaeR
CreB
KdpE
PhoP
CusR
YedW
BasR
QseB
FixJ
NtrCn
NarL
C
YFP-RR
Std of Δ FRET ratio (+PA)
CFP-RR
Std of Δ FRET ratio
0.9
0.6
0.4
0.2
0.1
0.09
0.08
0.07
0.06
0.05
0.04
0.03
0
D
YFP-RR
Without Phosphorylation
CFP-RR
Δ FRET ratio (527:475 nm)
- 0.05

Figure S4

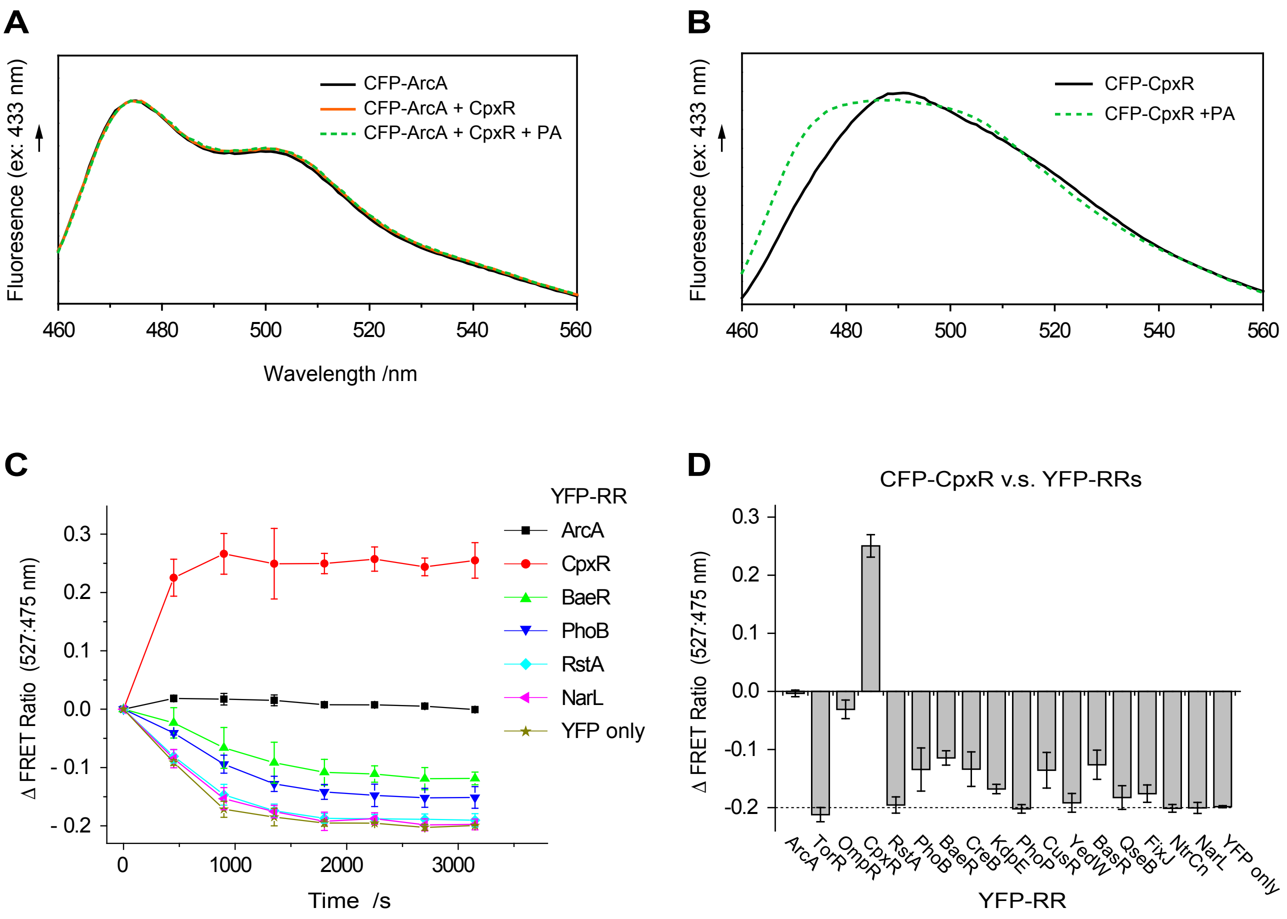
A
B
Fluoresence (ex: 433 nm)
CFP-ArcA
CFP-ArcA + CpxR
CFP-ArcA + CpxR + PA
Wavelength /nm
CFP-CpxR
CFP-CpxR +PA
C
D
YFP-RR
ArcA
CpxR
BaeR
PhoB
RstA
NarL
YFP only
Δ FRET Ratio (527:475 nm)
Time /s
CFP-CpxR v.s. YFP-RRs
ArcA TorR OmpR CpxR RstA PhoB BaeR CreB KdpE PhoP CusR YedW BasR QseB FixJ NtrCn NarL YFP only
YFP-RR
